# An unusual presentation of Bell's palsy: A case report and review of literature

**DOI:** 10.4103/0974-2700.40574

**Published:** 2008

**Authors:** Anna McFarlin, Bradley Peckler

**Affiliations:** 1 Center for Advanced Clinical Skills Lab, College of Medicine, University of South Florida, Tampa, FL, USA; 2 Department of Emergency Medicine, University of South Florida, Tampa, FL, USA

**Keywords:** Bells Palsy, retinal detachment

## Abstract

In clinical medicine there may be times when clinical conditions manifest differently both when they present individually or concomitantly. Such scenarios warrant a broader differential diagnosis with thorough investigations. We present one such case of a patient of Bell's palsy with unexplained eye pain on the ipsilateral side. The patient had a chronic retinal detachment which became worse due to the concomitant Bell's palsy.

A principle of Osler is that a constellation of symptoms is caused by one disease process. There are occasionally presentations that do not adhere to this principle. We present a classic presentation of a Bell's palsy with unexplained eye pain on the ipsilateral side. The patient had a chronic retinal detachment which became worse concomitantly with the Bell's palsy. While the two entities are unrelated it did create a large differential diagnosis requiring an extensive evaluation. We present the case report and a review of the diagnosis and management of Bell's palsy.

## CASE REPORT

A 47-year-old African-American man walked into the Emergency Department complaining of right-sided facial numbness and weakness. Before bed the previous night, the patient noted that fluid dripped out of the right side of his mouth while drinking. He then reawakened in the morning to right-sided facial droop, inability to close right eye and numbness to the right side of his face. He denied any numbness, tingling, or weakness in his extremities. On review of systems, he noted slight pain behind his right eye intermittently for the past few days and noted that he had developed a floater in his right eye 5 days ago. He denied recent cold sores, ear discharge, or recent trauma; however, he noted flu-like symptoms about 1-2 weeks ago. He also denied any changes in hearing or ear pain. The patient noted no previous medical history, however had not seen a doctor in more than 20 years. He denied any prior surgeries. He is a smoker but denied alcohol or illicit drug use.

On exam, the patient was in no acute distress and in no obvious discomfort. He was hypertensive with an initial blood pressure of 182/108. His speech was slightly slurred. He was alert and oriented times four. His skin was warm and dry with no apparent rashes or lesions. No nystagmus was noted. He demonstrated an inability to close his eyelid as well as generalized weakness of his right face including his forehead. His pupils were 3 mm, equal, round, and reactive. Extraocular movements were intact. Corrected visual acuity was 20/30 OD, OS, and OU. The fundus was not visualized on the right while the left appeared normal. No ulcerations were noted in his ears. The remainder of his exam including a thorough neurologic exam was unremarkable.

All diagnostic tests were essentially normal, including electrocardiogram and labs. Magnetic resonance imaging and magnetic resonance angiography of head and neck demonstrated no acute findings. Vascular tortuosity was noted, consistent with longstanding hypertension.

## DISCUSSION

Initial differential diagnosis for this patient included: stroke, Bell's palsy, vascular disease including carotid dissection, retinal detachment, herpes zoster ophthalmicus, middle ear lesions including Ramsey-Hunt syndrome, Human immunodeficiency virus, Lyme disease, Guillain-Barré syndrome, ocular lymphoma and vitreous hemorrhage, or inflammation. However, none of these diagnoses could explain the patient's facial paralysis and eye pain. Once imaging studies revealed no acute pathology and ophthalmologic testing revealed lattice degeneration and retinal detachment, it became clear that this patient had Bell's palsy with an incidental, likely chronic but only recently symptomatic retinal detachment. Upon thorough review of the literature, no cases were noted suggesting any link between the presentations of these separate processes. Once this patient's ocular symptoms were accounted for by the retinal detachment, his neurologic symptoms represented a classic presentation of seventh nerve palsy. His course was significant for complete recovery of facial nerve function on acyclovir and prednisone in addition to successful surgical correction of his retinal detachment.

Bell's palsy is a common neurologic disorder often inciting significant concern in patients and triggering stroke alerts in emergency departments. It is the most common cause of acute facial paralysis. According to one study, 68% of acute peripheral facial palsy were classified as Bell's palsy.[1] Incidence of Bell's palsy oscillates between 11 and 40 cases per 100,000 inhabitants per year, with 1 in 60 being affected in their lifetime.[2,3] Conventional wisdom states that incidence of Bell's palsy is higher in diabetic and pregnant patients; however, some recent studies dispute this.[4,5]

Bell's palsy is believed to be caused by inflammation of the facial nerve at the geniculate ganglion, which lead to compression and possible ischemia and demyelination.[6] Bell's palsy is a peripheral facial nerve palsy involving the lower motor neurons. A lower motor neuron lesion causes weakness of all the muscles of facial expression. The angle of the mouth falls, weakness of frontalis occurs, and eye closure is weak. With an upper motor neuron lesion, such as that seem acute stroke, the frontalis is spared, normal furrowing of the brow is preserved, and eye closure and blinking are not affected.[3]

Although Bell's palsy is often referred to as idiopathic, the cause can be infectious in nature. Herpes simplex virus activation has become widely accepted as the likely cause of Bell's palsy in most cases. Polymerase chain reaction DNA testing now verifies the notion of axonal spread and multiplication of a reactivated neuron-tropic virus leading to inflammation, demyelination, and palsy.[7,8] Other infectious causes of Bell's palsy include, but are not limited to Lyme disease, herpes zoster, cytomegalovirus, Epstein-Barr virus, adenovirus, rubella, mumps, influenza B, and coxsackievirus.[9]

Patients with Bell's palsy complain of sudden facial weakness (over the course of a few hours), difficulty with articulation, inability to keep an eye closed, or inability to keep food or drink in the mouth on one side. The seventh cranial nerve also serves other functions; the patient may have variable degrees of dry eye, metallic taste of the mouth, and facial pain commonly around the ear. Depending upon the site of the lesion, there may be associated impairment of taste, lacrimation, or hyperacusis. It is not uncommon for patients to have ipsilateral facial sensory impairment suggestive of ipsilateral trigeminal neuropathy such as in this particular patient. Such ipsilateral sensory loss in the setting of Bell's palsy has usually been attributed not to neuropathy, but to abnormal perception based on droopy facial muscles, skin, and associated tissue.[10]

The examination is notable for unilateral weakness of the face involving the forehead. The facial creases and nasolabial fold disappear, the forehead unfurrows, and the corner of the mouth droops. The eyelids will not close and lower lid sags. On attempted closure of the eyelid, the eye rolls upward (Bell's phenomenon).[6] Tear production decreases; however, the eye may appear to tear excessively because of loss of lid control allowing tears to spill freely from the eye. There may be a positive Hitselberger sign, a decreased sensation along the external acoustic meatus. Other cranial nerves are normal.

The diagnosis of Bell's palsy is essentially one of exclusion. While as previously described it is the most common cause of acute facial paralysis, its presentation is similar to potentially devastating events such as cerebrovascular accident (CVA) and tumor. No readily available laboratory test or imaging can verify the diagnosis of Bell's palsy. Key points to differentiate Bell's palsy from other etiologies include a rapid onset and progressive course, diffuse facial nerve involvement, normal reflexes, lack of vesicular lesions. Laboratory testing is usually not indicated unless the patient is at high risk for Lyme disease. Imaging is often warranted if other diagnoses are being considered, especially in the setting of an atypical presentation including insidious onset and forehead sparing.[10] Electrodiagnostic tests may aid in determining prognosis however are not typically indicated in patients who have a typical course.

Treatment for Bell's palsy is etiology-driven if a specific case is identified. Most patients with Bell's palsy recover without treatment 71% achieve complete recovery, 84% achieve near normal function.[11] Commonly employed, noncontroversial treatment modalities for Bell's palsy include eye patching and lubrication to protect the cornea from drying and abrasion secondary to poor lid closure and reduced tearing.

Controversy remains regarding the therapeutic effectiveness of steroids and acyclovir. According to the Cochrane Reviews, the available evidence from randomized controlled trials does not show significant benefit from treating Bell's palsy with steroids alone.[12] Upon completing their own systematic review, The American Academy of Neurology found insufficient evidence in class I studies to definitively establish the efficacy of steroid treatment. Nevertheless, based upon pooled results of class I and class II studies and a relatively benign side-effect profile, they concluded that steroids are safe and probably effective in improving facial functional outcomes in patients with Bell's palsy.[11] Treatment with corticosteroids should begin within 5 days (earlier if possible) after the onset of palsy and should only be used in the first 7 days.

Treatment with antivirals seems logical in Bell's palsy as the etiology is often viral. The American Academy of Neurology considers acyclovir safe and possibly effective in improving functional outcomes.[11] However, a recent double-blind, placebo-controlled, randomized study demonstrated no evidence of a benefit of acyclovir given alone or an additional benefit of acyclovir in combination with prednisolone.[13]

Other proposed treatments with little evidence to support them include Methylcobalamin, hyperbaric oxygen, facial retraining, botulinum toxin, transcutaneous electrical stimulation, and acupuncture.[3]

## CONCLUSION

Sir Arthur Conan Doyle wrote; “It is an old maxim of mine that when you have excluded the impossible, whatever remains, however improbable, must be the truth.”[14] This unusual case presented an interesting diagnostic dilemma and extensive work up. Bell's palsy and retinal detachment are common pathology but have no relation. It is important that providers consider unusual symptoms of common presentations and need to investigate them further if they do not fit into the normal symptomology.
